# *Balantidium grimi* n. sp. (Ciliophora, Litostomatea), a new species inhabiting the rectum of the frog *Quasipaa spinosa* from Lishui, China

**DOI:** 10.1051/parasite/2018031

**Published:** 2018-05-28

**Authors:** Weishan Zhao, Can Li, Dong Zhang, Runqiu Wang, Yingzhen Zheng, Hong Zou, Wenxiang Li, Shangong Wu, Guitang Wang, Ming Li

**Affiliations:** 1 Key Laboratory of Aquaculture Disease Control, Ministry of Agriculture, and State Key Laboratory of Freshwater Ecology and Biotechnology, Institute of Hydrobiology, Chinese Academy of Sciences, 430072 Wuhan PR China; 2 Hubei Key Laboratory of Animal Nutrition and Feed Science, Wuhan Polytechnic University, 430023 Wuhan PR China; 3 University of the Chinese Academy of Sciences, 100049 Beijing PR China; 4 Animal Husbandry and Aquaculture Station, Agriculture Forestry Animal Husbandry and Aquaculture Bureau of Guye District of Tangshan City, 063100 Tangshan PR China

**Keywords:** *Balantidium grimi*, ciliate, new species, *Quasipaa spinosa*, China

## Abstract

*Balantidium grimi* n. sp. is described from the rectum of the frog *Quasipaa spinosa* (Amphibia, Dicroglossidae) from Lishui, Zhejiang Province, China. The new species is described by both light microscopy (LM) and scanning electron microscopy (SEM), and a molecular phylogenetic analysis is also presented. This species has unique morphological features in that the body shape is somewhat flattened and the vestibulum is “V”-shaped, occupying nearly 3/8 to 4/7 of the body length. Only one contractile vacuole, situated at the posterior body, was observed. The phylogenetic analysis based on SSU-rDNA indicates that *B.* *grimi* groups together with *B.* *duodeni* and *B.* *entozoon*. In addition, the genus *Balantidium* is clearly polyphyletic.

## Abstract (fr)

*Balantidium grimi* n. sp. est décrit à partir du rectum de la grenouille *Quasipaa spinosa* (Amphibia, Dicroglossidae) de Lishui, province de Zhejiang, Chine. La nouvelle espèce est décrite à la fois par microscopie photonique et électronique à balayage, et une analyse phylogénétique moléculaire est également présentée. Cette espèce possède des caractéristiques morphologiques uniques en ce que la forme du corps est quelque peu aplatie et que le vestibule est en forme de « V » et occupe près de 3/8 à 4/7 de la longueur du corps. Une seule vacuole contractile, située au niveau du corps postérieur, a été observée. L’analyse phylogénétique basée sur la petite sous-unité de l’ADNr indique que *B.* *grimi* forme un groupe avec *B.* *duodeni* et *B.* *entozoon*. De plus, le genre *Balantidium* est clairement polyphylétique.

## 1 Introduction

The genus *Balantidium* Claparède & Lachmann, 1858 consists of many species inhabiting the digestive tract in a wide number of hosts from both invertebrate and vertebrate animals as endocommensals. They are generally considered harmless, but factors depressing the resistance of the host enable them to invade the mucosa and cause ulceration. The representatives of *Balantidium* have some common morphological features: cell body sacciform or slightly elongated in shape, and completely covered with cilia forming dense longitudinal rows [21]. To our knowledge, 31 amphibian balantidial species have been reported so far (lists in Li et al. [20]).

To date, 27 valid species have been reported in anuran amphibians, including *B. amygdalli* Bhatia & Gulati, 1927 [3], *B. aurangabadensis* Shete & Krishnamurthy, 1984 [34], *B. bicavata* Bhatia & Gulati, 1927 [3], *B. claperedei* Mahoon & Khan, 1986 [22], *B. corlissi* Shete & Krishnamurthy, 1984 [34], *B. cyanophlycti* Shete & Krishnamurthy, 1984 [34], *B. duodeni* Stein, 1867 [36], *B. elongatum* Stein, 1867 [36], *B. entozoon* Ehrenberg, 1838 [9], *B. falciformis* Walker, 1909 [40], *B. ganapatii* Shete & Krishnamurthy, 1984 [34], *B. giganteum* Bezzenberger, 1904 [2], *B. gracile* Bezzenberger, 1904 [2], *B. helenae* Bezzenberger, 1904 [2], *B. honghuensis* Li et al., 2013 [18], *B. kirbyi* Rodriguez, 1939 [31], *B. megastomae* Shete & Krishnamurthy, 1984 [34], *B. mininucleatum* Shete & Krishnamurthy, 1984 [34], *B. ranae* Shete & Krishnamurthy, 1984 [34], *B. ranarum* Ghosh, 1921 [10], *B. rotundum* Bezzenberger, 1904 [2], *B. sinensis* Nie, 1935 [24], *B. singaporensis* Khan & Ip, 1986 [16], *B. sushilii* Ray, 1932 [30], *B. tigrinae* Shete & Krishnamurthy, 1984 [34], *B. vanensis* Senler & Yildiz, 2000 [33] and *B. xenopi* Puytorac & Grain, 1965 [28]. Five other balantidial species were found in urodele amphibians, including *B. amblystomatis* Jírovec, 1930 [15], *B. andianusis* Li et al., 2008 [20], *B. elongatum* Stein, 1867 [36], *B. rayi* Pal & Dasgupta, 1978 [25] and *B. tylototritonis* Pal & Dasgupta, 1978 [25]. Among the aforementioned species, 3 balantidial species inhabiting amphibians were first discovered and named in China. *B. andianusis* was reported in the Chinese giant salamander, *Andrias davidianus* [20]; *B. sinensis* was described from 2 species of anuran amphibians and 1 urodele amphibian, *R. nigromaculata*, *R. plancyi* [24] and *A. davidianus* [20], respectively, and *B. honghuensis* was found in *R. nigromaculata* [18].

Although many amphibian *Balantidium* species have been reported, few molecular data are available at present (only two species *B. entozoon* and *B. duodeni* have corresponding SSU-rDNA sequences in NCBI). Even less is known about phylogenetic relationships between different balantidial groups inhabiting different hosts (such as fishes, amphibians, mammals, etc.).

In the present study, a new *Balantidium* species inhabiting *Quasipaa spinosa* is described based on detailed light and scanning electron microscopy observation. This is also the first record of *Balantidium* species in the digestive tract of *Quasipaa spinosa*. Phylogenetic analysis based on SSU-rDNA was also carried out to reveal the relationships among *Balantidium* species as well as different clades of Trichostomatia.

## 2 Materials and methods

### 2.1 Specimen collection and identification

The frogs used for this study were captured from Lishui City (27°25′–28°57′ N, 118°41′–120°26′ E), Zhejiang Province, southeast China in August, 2017. We obtained permits allowing us to capture and sacrifice these specimens. The frogs were transported alive to the laboratory, then all frog samples were anesthetized and dissected as soon as possible, the luminal contents of recta, intestines and duodena were collected respectively into different Petri dishes, and examined with the help of a stereomicroscope (Leica S8AP0, Germany). The ciliates were collected with Pasteur micropipettes and washed twice in 0.65% NaCl solution.

### 2.2 Light microscopy

Some specimens were fixed in 5% formalin for 10 min and soaked for about 30 min in 10% glycerin alcohol in a concave slide; the remaining specimens were fixed in Bouin’s fluids and stained with a Protargol method [11]. Specimens were observed, measured and photographed using a microscope (Olympus BX53, Japan). All measurements are in micrometers.

### 2.3 Scanning electron microscopy

The fully washed specimens were fixed in 2.5% glutaraldehyde in 0.2 M PBS (pH 7.4) on a clean glass slide (1 cm × 1 cm), which was previously treated with 0.1% poly-L-Lysine and dried completely in the air at room temperature. After being washed with PBS 3 times, they were post-fixed in 1% osmium tetroxide at 4°C for 1 h, followed by serial dehydration in acetone and critical point drying using the HCP-2 critical point dryer (Hitachi Science Systems, Japan). Subsequently, the glass slide was mounted on an aluminum-stub using a double-sided adhesive tape and sputter-coated with a thin layer of gold in IB-3 ion coater (Eiko Engineering, Japan), before observation and photography using a Quanta 200 SEM (FEI, Netherlands).

### 2.4 Extraction of genomic DNA and PCR amplification

About 50 individuals were harvested, suspended in lysis buffer (10 mM Tris-HCl, pH 8.0; 1 M EDTA, pH 8.0; 0.5 % sodium dodecyl sulfate; 60 µg/mL proteinase K), and incubated at 55°C for 12–20 h. DNA was extracted using a standard phenol/chloroform method, precipitated with ethanol, and resuspended in TE buffer. Polymerase chain reaction (PCR) amplifications were carried out using forward primer (5’-AACCTGGTTGATCCTGCCAGT-3’) and reverse primer (5’-TGATCCTTCTGCAGGTTCACCTAC-3’) [23]. The following cycling conditions included 5 min initial denaturation at 94°C; 35 cycles of 30s at 95°C, 1 min at 56-60 °C, and 1-2 min at 72°C; with a final extension of 10 min at 72°C. The PCR products were isolated using 1% agarose gel electrophoresis and purified using the Agarose Gel DNA Purification Kit (TaKaRa Biotechnology, Dalian, Japan). The amplified fragment was cloned into a pMD^®^18-T vector (TaKaRa Biotechnology, Dalian) and sequenced in both directions using M13 forward and reverse primers on an ABI PRISM^®^ 3730 DNA Sequencer (Applied Biosystems, USA). The SSU rRNA gene sequence of *B. grimi* was deposited in GenBank with accession number MG837094.

### 2.5 Phylogenetic analysis

Besides the SSU-rDNA sequence of *B. grimi* that we obtained in this study, other litostomatean sequences were retrieved from the GenBank/EMBL databases (Table 1). The sequence of *Nyctotheroides deslierresae* was used as the outgroup. The secondary structure-based SSU-rRNA sequence alignment of Litostomatea downloaded from the SILVA ribosomal RNA gene database project (https://www.arb-silva.de/) [29] was used as the “seed” alignment to build a profile Hidden Markov Model (HMM) using HMMER Package, version 3.1. Then the HMM profile obtained was used to create an alignment of the 40 sequences using Hmmalign within the package. The masked regions that could not be aligned unambiguously were removed from the initial alignment using MEGA 6.0 [39]. A GTR+I+G model was selected as the best model by the program jModelTest 2.1.10 [8] based on the AIC criterion, which was used for both Maximum Likelihood (ML) and Bayesian (BI) inference analysis. An ML tree was constructed with the RaxML program [35]. The reliability of internal branches was assessed using the non-parametric bootstrap method with 1,000 pseudoreplicates. A Bayesian analysis performed with MrBayes v3.2.6 [32] was run for 1,000,000 generations sampling every 1,000 generations. All trees below the observed stationary level were discarded as a burn-in of 25% of the generations.

**Table 1:** List of sequences from GenBank/EMBL databases used for phylogenetic analysis.

Species	GenBank/EMBL accession number	Reference
Trichostomatia		
Vestibuliferida		
*Balantidium polyvacuolum*	KJ124724	Li et al. [19]
*Balantidium ctenopharyngodoni*	GU480804	Li et al. [19]
*Balantidium entozoon*	EU581716	Grim and Buonanno [12]
*Balantidium duodeni*	KM057846	Chistyakova et al. [7]
***Balantidium grimi***	MG837094	present study
*Balantioides coli* (syn. *Balantidium coli*)	AM982723	Ponce-Gordo et al. [27]
	AM982722	Ponce-Gordo et al. [27]
*Dasytricha ruminantium*	U57769	Wright and Lynn [41]
*Isotricha intestinalis*	U57770	Wright and Lynn [41]
*Isotricha prostoma*	AF029762	Strüder-Kypke et al. [38]
*Helicozoster indicus*	AB794981	Ito et al. [14]
*Latteuria media*	AB794983	Ito et al. [14]
*Latteuria polyfaria*	AB794982	Ito et al. [14]
*Paraisotricha minuta*	AB794984	Ito et al. [14]
*Paraisotricha colpoidea*	EF632075	Strüder-Kypke et al. [37]
*Buxtonella sulcata*	AB794979	Ito et al. [14]
Macropodiniida		
*Amylovorax dehorityi*	AF298817	Cameron et al. [4]
*Amylovorax dogieli*	AF298825	Cameron et al. [4]
*Bitricha tasmaniensis*	AF298821	Cameron et al. [4]
*Bandia cribbi*	AF298824	Cameron and O’Donoghue [5]
*Bandia deveneyi*	AY380823	Cameron and O’Donoghue [5]
*Polycosta turniae*	AF298818	Cameron et al. (unpublished)
*Macropodinium yalabense*	AF042486	Wright (unpublished)
*Macropodinium ennuensis*	AF298820	Cameron et al. [6]
Entodiniomorphida		
*Cycloposthium bipalmatum*	AB530165	Imai et al. (unpublished)
*Troglodytella abrassarti*	AB437347	Irbis et al. [13]
*Ophryoscolex purkynjei*	U57768	Wright and Lynn [42]
*Epidinium caudatum*	U57763	Wright and Lynn [42]
*Entodinium caudatum*	U57765	Wright et al. [43]
*Diplodinium dentatum*	U57764	Wright and Lynn [42]
*Polyplastron multivesiculatum*	U57767	Wright et al. [43]
*Eudiplodinium maggii*	U57766	Wright and Lynn [42]
Haptoria		
Haptorida		
*Dileptus* sp.	AF029764	Strüder-Kypke et al. [38]
*Homalozoon vermiculare*	L26447	Leipe et al. [17]
*Enchelys polynucleata*	DQ411861	Strüder-Kypke et al. [38]
*Spathidium stammeri*	DQ411862	Strüder-Kypke et al. [38]
*Didinium nasutum*	U57771	Wright and Lynn [41]
Pleurostomatida		
Amphileptus procerus*	AY102175	Zhu et al. (unpublished)
*Loxophyllum rostratum*	DQ411864	Strüder-Kypke et al. [38]
Armophorea		
Clevelandellida		
*Nyctotheroides deslierresae*	AF145353	Affa’a et al. [1]

* submitted as *Hemiophrys procera*, according to Strüder-Kypke et al. [37].

## 3 Results

Ninety-eight individuals of *Q. spinosa* were examined in the present study and 34 were found to be infected with *Balantidium* *grimi* (prevalence, 34.7%). These specimens were found mainly in the recta of frogs.

***Balantidiumgrimi* n. sp.**

urn:lsid:zoobank.org:act:84E00073-0D0C-4166-8D83-20BFCC43480E

**Type host:** *Quasipaa spinosa* David, 1875.

**Prevalence:** 34.7% (34 of 98) of *Q. spinosa* were infected.

**Type locality:** Lishui City (27°25′–28°57′N, 118°41′–120°26′E), Zhejiang Province, China.

**Infection site:** Rectum.

**Type material:** Holotype catalogued under No. IHB2017W005, paratype catalogued under No. IHB2017W006 with protargol stained and the rest of ciliates preserved in 100% alcohol (Nos. LS001-002), 2.5% glutaraldehyde (No. LS003) and Bouin’s fluids (Nos. LS004-LS006) have been deposited in Key laboratory of Aquaculture Disease Control, Ministry of Agriculture, Institute of Hydrobiology, Chinese Academy of Sciences, China.

**Etymology:** The new species was designated *Balantidium grimi* n. sp. in honor of the great contributions of Prof. J. Norman Grim to parasitic and symbiotic ciliates.

### 3.1 Morphology under light microscope

**Figure 1:**
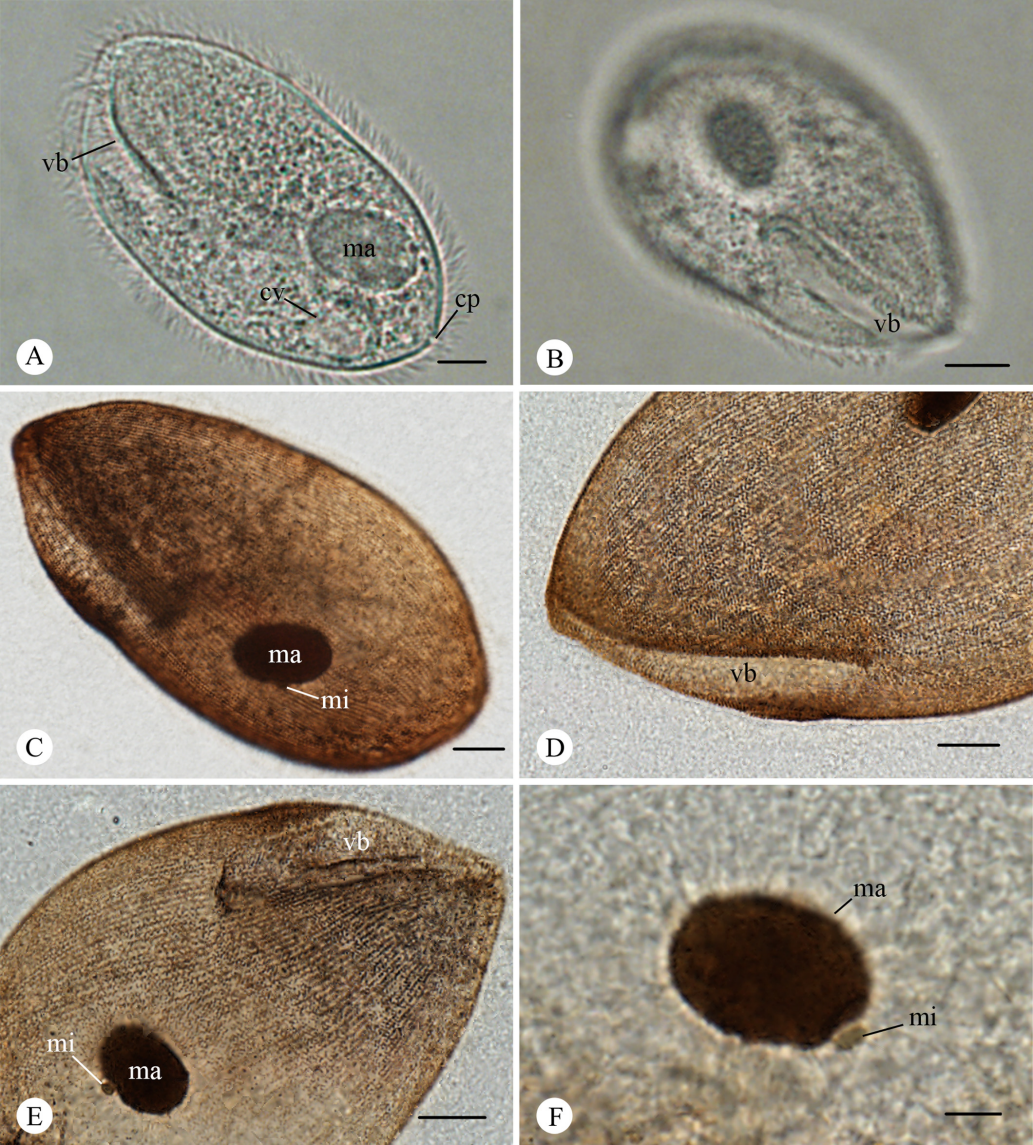
LM images of *B. grimi*. **A.** Specimens fixed in formalin (5%) and soaked in glycerine-alcohol (10%), showing the oval body shape, vestibulum (vb) and macronucleus (ma), a round contractile vacuole (cv) in the posterior and a cytoproct (cp) at the end of the body. Scale bar = 10 µm. **B.** Specimens fixed in formalin (5%) and soaked in glycerine-alcohol (10%), showing the long vestibulum (vb) surrounded by cilia. Scale bar = 10 µm. **C-F.** are protargol stained: **C.** showing the body shape, macronucleus (ma) and micronucleus (mi). Scale bar = 10 µm. **D.** showing the vestibulum and somatic kineties. Scale bar = 10 µm. **E.** showing the vestibulum (vb) and the oval macronucleus (ma) with a spherical micronucleus (mi) embedded in the middle. Scale bar = 10 µm. **F.** showing the relative position of macronucleus (ma) and micronucleus (mi). Scale bar = 5 µm.

**Figure 2:**
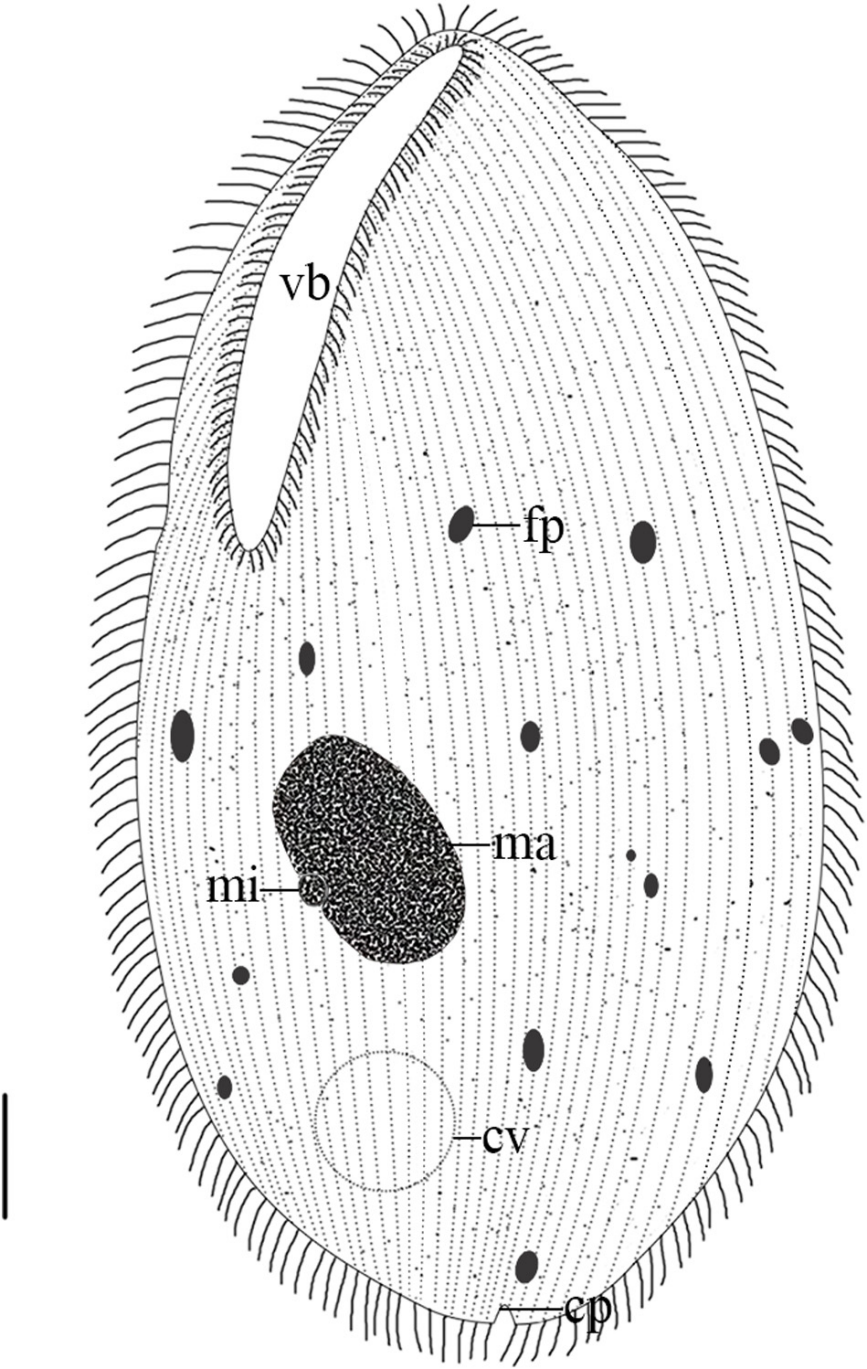
Schematic drawing of *B. grimi*, showing the general form and structures from the ventral-left view: vestibulum (vb), food particles (fp), macronucleus (ma), micronucleus (mi), contractile vacuole (cv) and cytoproct (cp). Scale bar = 10 µm.

Organism long-oval in shape (Figures 1A, C and 2), measuring 79.6-121.5µm ($\bar{\text{X}}$ = 96.5 µm; n = 30) in length and 43.6-83.6 µm ($\bar{\text{X}}$ = 57.8 µm) in width. Body partially flattened and thickly ciliated (Figures 1A, C and 2). The number of body kineties ranged from 93 to 125, oriented mostly parallel to the cell’s long axis. Of these, 41 to 59 were dorsal and 52 to 67 were ventral. Vestibulum “V”-shaped, 32.6-53.9 µm ($\bar{\text{X}}$ = 43.43 µm, n = 30) in length, accounted for 3/8 to 4/7 of the body length (Figures 1B, D, E and 2), and 3.9-5.9 µm ($\bar{\text{X}}$ = 4.7 µm, n = 30) in width. Macronucleus oval and lay obliquely almost near the middle of body (Figures 1C, E, F and 2), 20.0-29.2 µm ($\bar{\text{X}}$ = 24.1 µm, n = 30) in length and 12.4-19.3 µm ($\bar{\text{X}}$ = 16.0 µm, n = 30) in width. Micronucleus spherical or somewhat oval near the macronucleus (Figures 1C, E, F and 2), measuring approximately 2.2-2.9 µm ($\bar{\text{X}}$ = 2.5 µm, n = 13) in diameter. A distinct contractile vacuole situated at the posterior region of the body with 12.4-15.4 µm ($\bar{\text{X}}$ = 13.7 µm, n = 8) in diameter (Figures 1A and 2). A cytoproct present at the posterior end of the body (Figures 1A and 2). Detailed morphometric parameters are presented in Table 2.

**Table 2:** Morphometric light microscopic parameters of *B. grimi*.

Character	$\bar{\text{X}}$	M	Min	Max	SD	SE	CV(%)	N
Body length (Lb)	96.5	95.1	79.6	121.5	9.65	1.76	10.0	30
Body width	57.8	55.4	43.6	83.6	9.43	1.72	16.3	30
Vestibulum length (Lv)	43.4	44.0	32.6	53.9	4.43	0.81	10.2	30
Vestibulum width	4.7	4.7	3.9	5.9	0.44	0.08	9.4	30
Macronucleus length	24.1	24.4	20.0	29.2	2.11	0.38	8.8	30
Macronucleus width	16.0	16.1	12.4	19.3	1.88	0.34	11.8	30
Micronucleus diameter	2.5	2.5	2.2	2.9	0.21	0.06	8.1	13
Contractile vacuole diameter	13.7	13.5	12.4	15.4	1.08	0.38	7.9	8
Lb/Lv	2.2	2.3	1.7	2.7	0.23	0.04	10.5	30
Number of kineties on the left	51.2	51	41	59	5.56	1.85	10.9	9
Number of kineties on the right	61.1	62	52	67	5.21	1.74	8.5	9

Measurements are in µm. $\bar{\text{X}}$: arithmetic mean, M: median, Min: minimum, Max: maximum, SD: standard deviation, SE: standard error, CV: coefficient of variation, N: number of individuals investigated.

### 3.2 Morphology under scanning electron microscope

**Figure 3:**
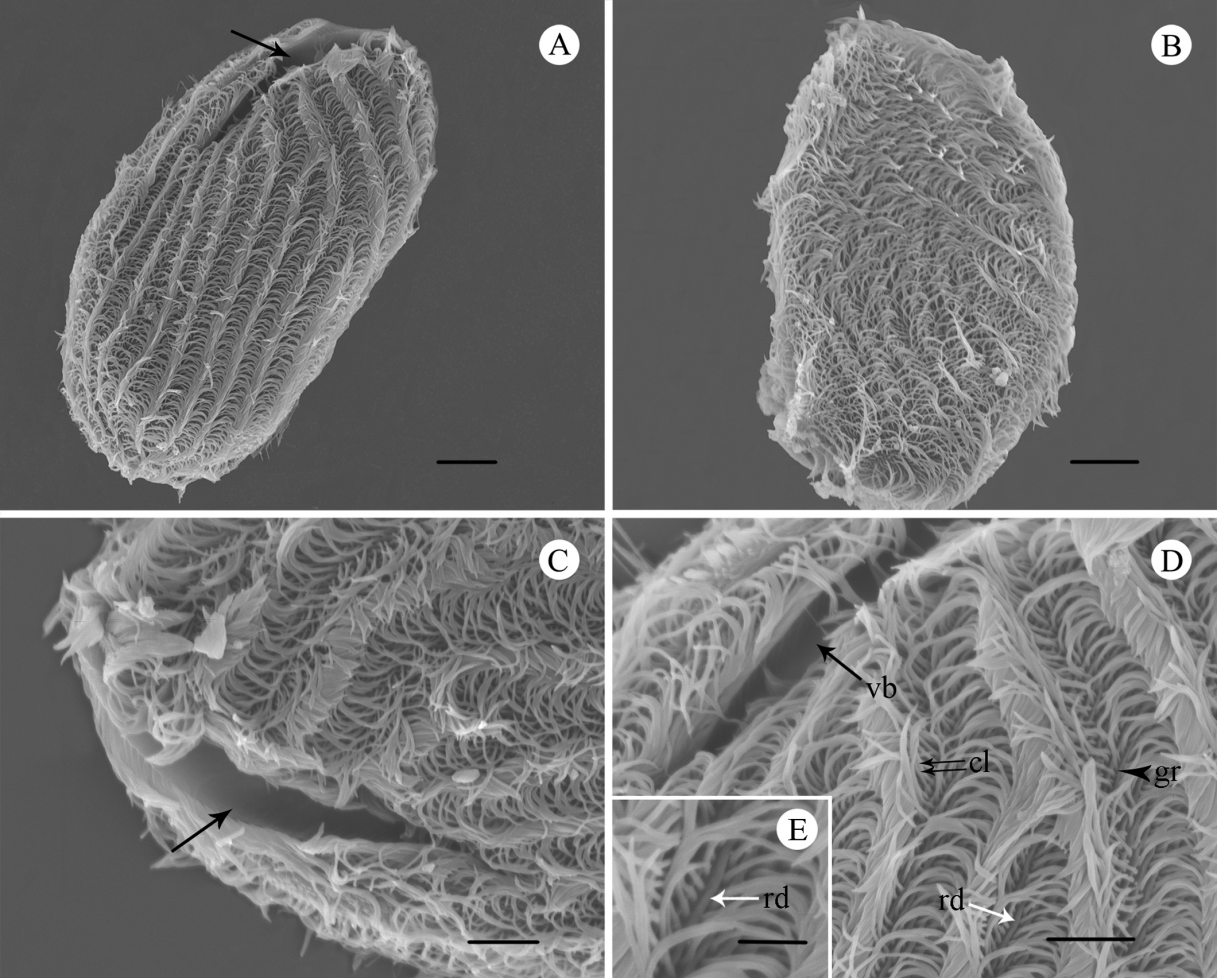
SEM images of *B. grimi*. **A.** Overview of the ventral-left side (oral side), showing the general form, vestibulum (arrow) and uniformly arranged cilia. Scale bar = 10 µm. **B.** Overview of the right side, showing the body surface is partially flattened and thickly ciliated. Scale bar = 10 µm. **C.** Ventral-left view of the “V”-shaped vestibulum (arrow). Scale bar = 5 µm. **D.** The left anterior area of ciliate, showing the vestibulum (vb), an interkinetal ridge (rd), the groove (gr) and the cilia (cl) extending from grooves and are close to one another. Scale bar = 5 µm. **E.** Selected enlargement of Figure 3D, showing a ridge (rd) between cilia. Scale bar = 2 µm.

*B. grimi* is thickly ciliated, but with uniform arrangement on the cell surface (Figures 3A, B). Regular beat patterns of cilia that look like “waves” make the cell move smoothly (Figure 3A). The “waves” and ridges formed an angle ranging from 0° (at the posterior) to 60° (at the anterior) (Figures 3A, C, D). Numerous cortical grooves arranged alternately with cortical ridges, which are parallel to the longitudinal axis of the body (Figure 3D). The cilia originate within grooves and are quite close together; those in Figure 3D are about 0.62 µm apart.

### 3.3 Phylogenetic analysis

**Figure 4:**
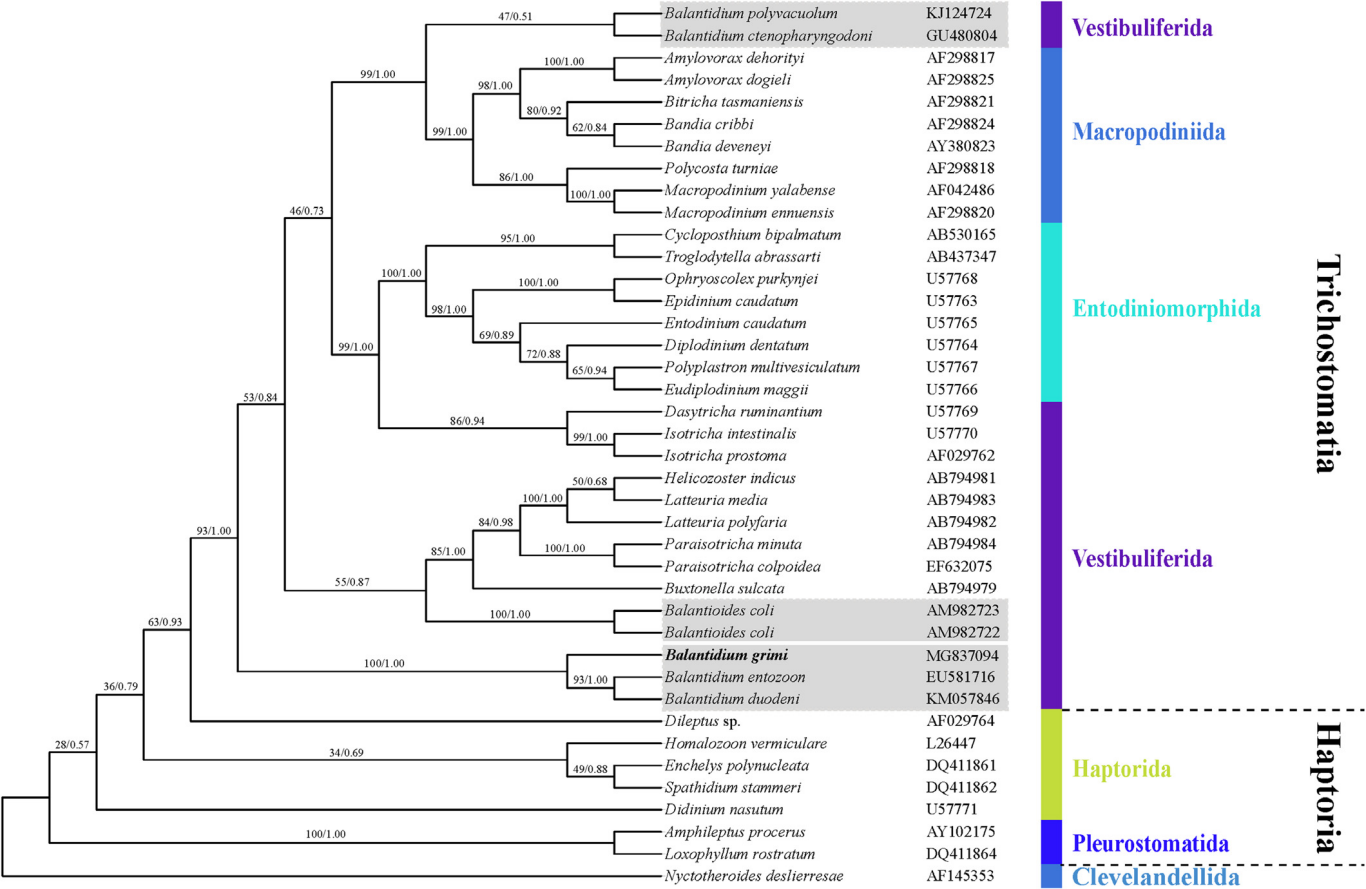
Phylogenetic relationships of the SSU-rRNA sequences of *B. grimi* marked in bold and other Trichostomatia species showing the position of *B. grimi* inferred by maximum likelihood method and Bayesian algorithm. The trees were rooted using the sequence of *Nyctotheroides deslierresae* as the outgroup taxa. Numbers at nodes indicate bootstrap percentage and posterior probability, respectively. The sequences corresponding to species of the genus *Balantidium* are shadowed.

The sequenced SSU-rRNA gene of *B. grimi* is 1,640 bases in length and the guanine-cytosine (GC) content is 42.26%. The topologies of our phylogenetic trees generated using MrBayes and PhyML algorithms are totally accordant (Figure 4). Species of the family Balantidiidae are separated into three clades. *B. grimi* grouped together with *B. duodeni* and the type species of the genus, *B. entozoon*, and form the first clade whose hosts are anuran amphibians (100% ML, 1.00 BI). *B.* *polyvacuolum* and *B. ctenopharyngodoni* form the second balantidial clade inhabiting fish hosts. The third group consisted of two isolates of *B. coli*, which were reported from many mammalian hosts, including pigs and humans.

## 4 Discussion

A new *Balantidium* species inhabiting Chinese anuran amphibians *Quasipaa spinosa* is recorded herein. To our knowledge, this is the first report of *Balantidium* species in *Q. spinosa*.

*B. grimi* is quite unique considering its remarkably flattened body and conspicuous slit-shaped vestibulum, which can distinguish it from other *Balantidium* species [7,12,21]. *B. grimi* resembles *B.entozoon*, *B. duodeni*, *B. helenae* and *B. sinensis* in some aspects. For example, *B. grimi* shares a similar Lv/Lb value with *B. duodeni* [7]. But in terms of body forms and dimensions, these two balantidial species could easily be discriminated from each other. As to the shape and dimension of the macronucleus, as well as the position of the contractile vacuole, *B. grimi* somewhat resembles *B. helenae* [33], but the latter species possesses a remarkable “knob” at the posterior end. Comparisons were also made between *B. grimi* and *B. sinensis* inhabiting the Chinese giant salamander *Andrias davidianus* [20] as well as *B. entozoon*, the type species of the genus *Balantidium* [12]. Detailed comparisons of morphometric parameters among corresponding *Balantidium* species are presented in Table 3.

**Table 3:** Comparison of body length (Lb), vestibulum length (Lv) and the ratio of vestibulum length and body length (Lv/Lb) between *B. grimi* and four *Balantidium* species.

Species	Host	Body length (Lb)			Vestibulum length (Lv)			Lv/Lb		
				
		$\bar{\text{X}}$	Min	Max	$\bar{\text{X}}$	Min	Max	$\bar{\text{X}}$	Min	Max
*Balantidium entozoon*	*Rana esculenta*	83.3	60.0	129.0	27.7	20.0	34.0	0.33	0.19	0.48
*Balantidium duodeni*	*Rana temporaria*	128.6	111.6	156.9	56.3	44.2	76.7	0.44	0.40	0.60
*Balantidium helenae*	*Rana ridibunda*	88.9	62.5	112.5	33.2	25.0	50.0	0.37	0.29	0.52
*Balantidium sinensis*	*Andrias davidianus*	138.3	120.0	158.4	47.0	40.8	52.8	0.34	0.30	0.44
*Balantidium grimi*	*Quasipaa spinosa*	96.5	79.6	121.5	43.4	32.6	53.9	0.44	0.37	0.58

Measurements are in µm. $\bar{\text{X}}$: arithmetic mean, Min: minimum, Max: maximum.

According to the molecular phylogenetic analysis, the order Macropodiniida ciliates is closely related to fish balantidial species [14,19]. The affinity implies that macropodiniids may have been the result of separate invasions of terrestrial hosts by ciliates initially associated with aquatic hosts [19]. Macropodiniids, previously called “Australian clade”, possess similar oral cavities to some vestibuliferids that are bordered by somatic kineties and analogous ultrastructure to the Isotrichidae [5,21,37,38]. Moreover, the strong molecular support of Macropodiniida assemblage as a sister clade to the Balantidiidae (fish balantidia) also gives us an indication that Macropodiniida ought to be incorporated into the order Vestibuliferida, which also coincides with the viewpoint of former studies [5,14,19].

Our results show that the genus *Balantidium* is clearly polyphyletic and all *Balantidium* species are separated into three distinct clades, according to host specificity: fish balantidia (*B. ctenopharyngodoni* and *B. polyvacuolum*), amphibian balantidia (*B. grimi*, *B. entozoon* and *B. duodeni*), and balantidia from warm-blooded vertebrates (*Balantioides coli*) [7]. Pomajbíková et al. [26] has proposed a new genus *Neobalantidium* for the third group. However, it was recently suggested to reinstate the genus *Balantioides* as this taxon has been named for a long time [7]. Here, we accepted the generic name *Balantioides* to describe this group. As to the amphibian balantidia, our new species clustered with the other two species, *B. entozoon* and *B. duodeni* with maximum molecular supports. On this point, our results are consistent with those of Chistyakova et al. [7], but differ from those of Li et al. [19]. We suspect that the key reason for this disagreement is the quantity of introduced species used for phylogenetic analysis. The greater the number of related species studied, the greater the accuracy of the resulting phylogeny. Thus, more molecular information on *Balantidium* species from fishes and amphibians as well as reptiles is needed to clarify their phylogenetic relationships.

## Conflict of interest

The authors declare that they have no competing interests.
